# Gaze and viewing angle influence visual stabilization of upright posture

**DOI:** 10.1002/brb3.10

**Published:** 2011-09

**Authors:** KI Ustinova, J Perkins

**Affiliations:** Graduate Program in Physical Therapy, Herbert H. and Grace A. Dow College of Health Professions, Central Michigan UniversityMichigan 48859

**Keywords:** Eye movements, postural control, visual perception

## Abstract

Focusing gaze on a target helps stabilize upright posture. We investigated how this visual stabilization can be affected by observing a target presented under different gaze and viewing angles. In a series of 10-second trials, participants (*N* = 20, 29.3 ± 9 years of age) stood on a force plate and fixed their gaze on a figure presented on a screen at a distance of 1 m. The figure changed position (gaze angle: eye level (0°), 25° up or down), vertical body orientation (viewing angle: at eye level but rotated 25° as if leaning toward or away from the participant), or both (gaze and viewing angle: 25° up or down with the rotation equivalent of a natural visual perspective). Amplitude of participants’ sagittal displacement, surface area, and angular position of the center of gravity (COG) were compared. Results showed decreased COG velocity and amplitude for up and down gaze angles. Changes in viewing angles resulted in altered body alignment and increased amplitude of COG displacement. No significant changes in postural stability were observed when both gaze and viewing angles were altered. Results suggest that both the gaze angle and viewing perspective may be essential variables of the visuomotor system modulating postural responses.

## Introduction

Focusing gaze on a stationary target during standing helps minimize body oscillations and increase stability of upright posture. This mechanism is helpful in many situations, for example standing in a moving environment or on an uneven surface, or when in environments with conflicting sensory inputs. The efficiency of visual stabilization depends on many factors such as target size and location, viewing distance, visual acuity, and eye vergence (Paulus et al. 1984; Stoffregen 1985; Paulus et al. 1989; Previc and Neel 1995; Piponnier et al. 2009). What is less known is whether postural stability can be affected by viewing a target under different angular perspectives.

Indeed, the angle under which we observe our environment and objects located in it plays an essential role in motor performance**.** In literature, this angle is defined by two vectors, the first connecting the eye with the observed target, and the second formed by the line projected horizontally and straight ahead at eye level (Schmidt et al. 1993; Vaillancourt et al. 2006; Shieh and Lee 2007). Viewing a target under different angular perspectives modulates neural signal processing in multiple brain areas involved in planning and preparing movement (Baker et al. 1999; DeSouza et al. 2000; Bédard et al. 2008) and affects various parameters of postural and motor tasks performance. For example, standing and focusing gaze on a target presented above and below horizontal eye level has been reported to reduce oscillations of upright posture (Kapoula and Lê 2006). Similar to findings of postural control research, it has been shown that altering the visual angle affects a participant's estimation of distance to an object (Levin and Haber 1993; Gardner and Mon–Williams 2001) and the ability to apply a constant level of force to a load cell using feedback presented at different angles (Vaillancourt et al. 2006). Selecting a particular visual angle for a task has been shown to facilitate reading a book (Schmidt et al. 1993; Shieh and Lee 2007) and “improve task performance” (Sommerich et al. 2001). Thus, there is considerable evidence that altering the visual angle can influence postural and voluntary movement control. However, the mechanism of this effect is unclear.

As people move their eyes and bodies during normal daily activities they alter the position of their eyes in the orbits (gaze angle), the image projection on the eye retina as observed from different points of view (viewing angles), and head position—if viewing of an object requires eye movement amplitude beyond that achieved with eye movement alone. The contribution of each specific factor to the motor control and specifically to the visual stabilization of upright posture is unclear. Investigation of this question is important and can help our understanding of the mechanism underlying the visuomotor transformation for postural control.

In this study, we attempted to dissociate the components of the visual angle to allow investigation of the effect of gaze versus viewing angle on postural stability during quiet stance. Previous research (Ustinova et al. 2010) showed that manipulating the viewing angle in a virtual environment without eye movement altered participants’ performance of functional reaching for a target while standing. This leads us to hypothesize that viewing a target under different perspectives could influence postural stability as well.

From a practical standpoint, the results of the study could be used in simulated environments such as gaming, virtual rehabilitation for balance, and teleoperator training. In these environments, usually presented to participants on a screen or via “head-mounted display,” natural eye movements are limited (Sandor and Leger 1991; Ukai et al. 2001). Consequently, participants experience a conflict between visual information, perception, and eye position signals (Stoffregen et al. 2008). Thus, it is important to determine the best viewing perspective for postural stability or other accurate motor performance in these virtual environments.

## Methods

### Participants

Twenty females with age range of 23–52 years (29.3 ± 9 years), were recruited from the university community. The project received approval from the university Institutional Review Board (IRB). Participants had no known balance or motor impairments, perceptual problems, or other orthopedic and neurological conditions that would interfere with their ability to perform the experimental task. Visual acuity was assessed with a Snellen chart placed at 20 feet. All participants demonstrated adequate performance for the test with results ranging from 20/10 to 20/50 for the left and right eyes. Six participants used glasses or contact lenses for visual acuity correction.

### Experimental task

**Figure 1:**
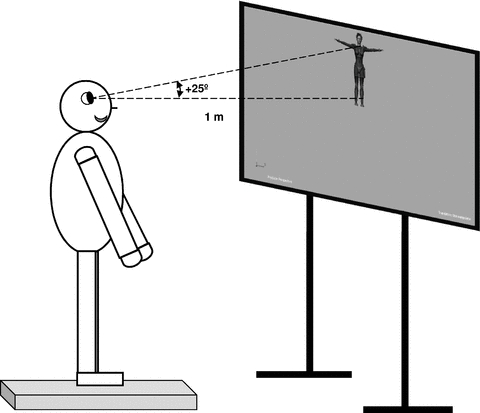
Participant standing in front of the screen and looking at the target presented at +25° gaze angle.

The experimental task consisted of standing quietly on bilateral force plate (Neurocom International Inc., Oregon, USA) in front of a 91×122 cm flat screen and viewing a target presented on this screen (Fig. 1). The screen was placed at 1-m distance from the participant's eyes. The target was the computer-generated character “Mia” standing in T-stance (upright with arms stretched out to the side). The character was created with the use of Autodesk MotionBuilder 7.0 software.

**Figure 2:**
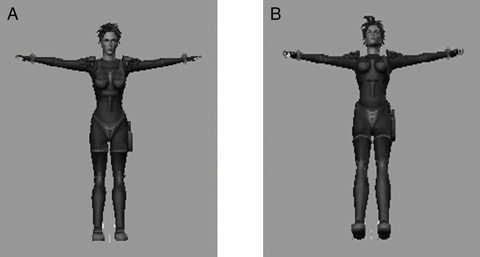
The target character “Mia” presented at 0° (left panel) and +25° (right panel) viewing angles.

In baseline trials (0°), the character was presented so that the cross formed by her arms and body in T-stance was at the participant's eye level. In other trials, the character was presented in a randomized order in a manner that required the participant to alter gaze angle, or viewing angle or both (Fig. 2). To change a gaze angle, the character was shifted vertically up or down on the screen (to create gaze angles of approximately +25° or −25° above and below eye level), while keeping the visual image constant. The viewing angle was manipulated by rotating the figure, thereby altering the character's apparent vertical body orientation, while maintaining its location in the middle of the screen (Fig. 2B). The character was presented as if leaning toward the participant (+25°) or away from the participant (−25°). In other trials, the character changed both position on the screen and vertical orientation simultaneously (gaze and viewing angle +25° or −25°), thereby creating a naturalistic visual perspective similar to the real-world situation of looking at a person from above or below. In addition to the character-manipulation, a set of trials was done without a character, and participants were asked to stand quietly looking at the gray screen in front of them.

Participants were asked to watch the character body without moving their head during the different experimental gaze and viewing angle conditions. Physiologically, viewing a target deviated from the eye level up to 25° does not require head movements. Each trial lasted 10 seconds and was repeated three times in each of the eight conditions for a total number of 24 trials.

### Data collection and analysis

Kinetic data from the bilateral force plate were collected, and the parameters of center of gravity (COG) displacement were analyzed. These parameters included the amplitude of the COG sagittal displacement, the surface area of the COG excursion, and maximum forward and backward angular displacements of the COG. The amplitude was computed as a deviation between maximum and minimum COG shifts. The surface area of the COG was calculated so that 95% of the COG displacements were inside the ellipsoid, formed by sagittal and frontal COG displacements. Angular displacement was calculated according to the following equation (Neurocom, Operator Manual, 2000):

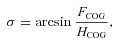
where F_COG_ is the sagittal displacement of the COG from the vertical line formed by lateral malleoli and the point equal to approximately 55% of participant's total height (H_COG_).The COG oscillations in the frontal plane were not analyzed as they are “less meaningful” for postural control during quiet stance in healthy individuals (e.g., Winter 1995). Overall, an increase of the COG oscillations was considered as reducing postural stability, while decreased COG oscillations were evidence of postural stabilization, or improved postural stability.

A mixed two-way Analysis of Variance (ANOVA) with appropriate *t*-test was used to analyze the influence of experimental condition (viewing, gaze, or gaze/viewing) and angle (0°, 25°, or −25°) on the COG parameters.

## Results

**Figure 3:**
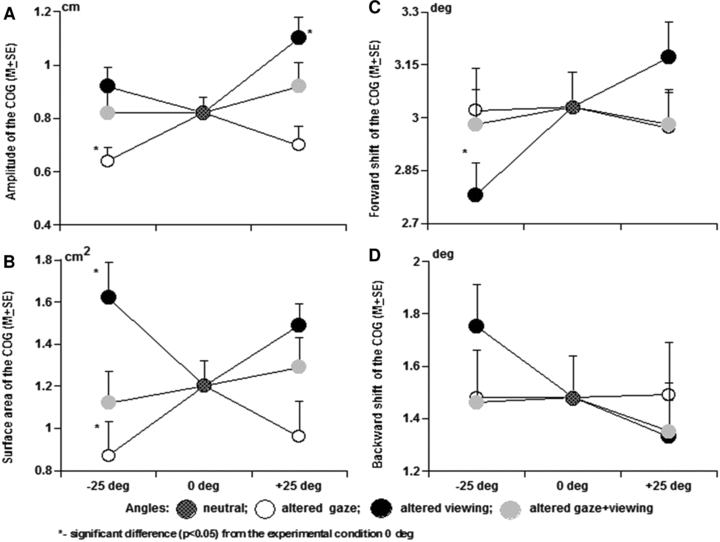
The averaged means (±SE) of the COG parameters: amplitude of displacement (A); surface area (B); maximum angular shift forward (C); and backward (D). The means identify the parameter in neutral condition (dashed circle) and their changes due to manipulation with gaze angle (open circles), viewing angle (black circles), and both gaze and viewing angle (gray circles) from −25° to +25°.

Manipulating the gaze, viewing, and both gaze and viewing conditions influenced upright postural stability in all participants with significant overall ANOVA effect (*F*_(4,114)_ = 4.25; *P* = 0.003). The averaged means (±SE) of the parameters characterizing the COG oscillations are presented in Fig. 3. There was a significant effect of experimental condition on amplitude of the COG displacement (*F*_(2,57)_ = 5.05; *P* = 0.009), surface area (*F*_(2,57)_ = 4.62; *P* = 0.014), and maximum forward COG shift (*F*_(2,57)_ = 3.04; *P* = 0.01). However, no differences between conditions were found in the maximum backward shift of the COG. Overall, the amplitude of the COG oscillations was decreased by 32% (from 1.09 to 0.82 cm, *P* = 0.012) when participants stood with gaze fixated on a target, presented at the neutral position (0^0^), with no difference found in the COG surface area, or forward and backward body alignment.

Altering angular presentation of the target did not result in any significant overall effects on COG parameters (e.g., amplitude of the COG displacement *F*_(2,114)_ = 0,69; *P* = 0.501).When gaze angle deviated from the neutral position, the following significant changes were found (Fig. 3A–D, open circles). Observing the character located below eye level (angle −25°) resulted in reducing the amplitude by 21% (from 0.82 to 0.64 cm, *P* = 0.031) and surface area by 27% (from 1.2 to 0.87cm^2^, *P* = 0.041) of the COG displacement. The same parameters had a nonsignificant tendency to reduce under gaze angle +25°. No difference was found in maximum forward and backward angular displacement with either gaze deviation.

Altering the viewing angle (Fig. 3A–D, black circles) increased the amplitude of postural oscillations by 34% (from 0.82 to 1.10 cm, *P* = 0.012) when participants viewed the character oriented under +25°, with a tendency for increased amplitude when the character was oriented in the opposite −25° down position. There was a significant increase in surface area by 42% (from 1.2 to 1.62cm^2^, *P* = 0.035) in the −25° viewing position, however. One finding of interest was that altering the viewing angle changed body alignment. When the character was viewed in the -25° presentation participants shifted their body backwards, with significantly decreased maximum forward displacement by 8% (from 3.03° to 2.78°, *P* = 0.013). Although not significant, a similar tendency was revealed when watching the character in +25°. Participants slightly leaned forward increasing the maximum forward and decreasing the maximum backward angular displacements, respectively.

Altering both gaze and viewing angles did not result in any significant changes in COG parameters. However a tendency for increased amplitude of the COG oscillations was observed.

## Discussion

### Basic findings

Overall results demonstrated that visual stabilization of upright posture was influenced by altering either gaze or viewing angles. Changing the gaze angle, so eyes either looked up or down, reduced the surface area and amplitude of postural oscillations. In contrast, presenting the character in different viewing angles, as if leaning toward or away from the participant, destabilized posture by altering the body alignment and increasing the amplitude and surface area of the COG displacement. No significant differences in parameters of the COG oscillations were observed when both gaze and viewing angles were altered together, although there was a tendency noted for an increase in postural oscillations, similar to that seen with changing viewing angle alone.

### Effect of gaze angle

An effect of gaze angle on postural stabilization was anticipated and consistent with the work of Kapoula and Lê (2006). They showed that depression or the elevation of the eyes of 15° up or down to watch a target placed at 2-m distance increased postural stability as compared with looking straight ahead. Physiologically, when looking straight ahead, the extraocular muscles that move the eyes in the orbits in the vertical plane are relaxed. Looking either up or down increases their activity. Proprioceptive feedback from these extraocular muscles modifies activity of the neck muscles through a chain of brainstem reflexes even when head is not moving (Andre´–Deshays et al. 1988; Andre´–Deshays et al. 1991; Corneil et al. 2004). Neck muscle activity is thought to be a powerful mediator of postural control (Kogler et al. 2000; Vuillerme and Rougier 2005), and could reduce body oscillations in our participants.

Postural reorganization could also be mediated by changes in the visual signal processing in the eye retina and particularly the peripheral part. Peripheral vision plays an important role in control of quiet standing in a relatively stationary or moving environment (Previc and Neel 1995; Berencsi et al. 2005). When swaying slightly in quiet stance, an individual observes the environment through a coherent available optic array (Gibson 1954; Lee and Lishman 1975). The velocity and structure of this optic array are not the same everywhere and are accommodated differently by central and peripheral parts of the eye retina (Brandt et al. 1973; Berthoz et al. 1975; Stoffregen 1985, 1986). When gaze is directed straight ahead, the velocity of optic flow increases with eccentricity, that is, angular deviation from the line corresponding to the direction of sway. The highest velocity optical transformations are found in the extreme visual periphery and this velocity increases as the visual stimulus approaches the observer. Higher velocity optical transformations are both more detectable and useful than the lower velocity transformations. Therefore, the peripheral retina appears to be more efficient than the central part in detection of posture-related stimuli in the optic flow, for example, those generated by supporting surface, (Stoffregen 1985). When our participants directed their gaze downward, a visible supporting surface (floor) appeared in their visual periphery, and the distance between eye and support was decreased compared to when they looked straight ahead. This induced sensitivity to the most informative optical transformations and could reduce postural oscillations. Although data collection was conducted in a relatively “dimly lit” environment, this effect of this peripheral visual effect could not be excluded.

It is also important to mention that an alternative point of view on the contribution of peripheral and central visual systems to postural control exists. Several studies showed an equal importance of both systems in maintenance of upright posture (Straube et al. 1994; Piponnier et al. 2009). If this is the case, postural sway reduction in gaze up and down conditions could be partially explained by head stabilization in our subjects. In healthy adults, postural control during standing and walking (Assaiante et al. 2005) can be organized in top-to-down manner, where the head serves as a frame of reference for upright stance. When looking up or down, our participants consciously minimized head motion. This head stabilization could simply cause stabilization of the entire body functioning as a closed kinetic chain, and as a result could reduce the amount of postural oscillations.

### Effect of viewing angle

The effect of altering the viewing angle on postural stability has not been investigated previously in a systematic way. This finding is consistent with the results of our previous study. That work showed that viewing a target under mid-range angles in a virtual environment increased involvement of the trunk and leg segments in arm transport during reaching while standing (Ustinova et al. 2010). As a result, participants reached further. This suggests that viewing perspective can be a variable influencing visual signals transformation not only for control of voluntary movements, but for postural control as well. There are a few potential mechanisms that can modify such transformation.

Induced postural oscillations, when seeing the character apparently leaning forward or away, might be altered perception of vertical position. Distortion of the visual environment alters perception of the body's vertical orientation within it (Carriot et al. 2008; Keshner and Kenyon 2009). Consequently, it results in postural reorganization and shifting of the body away from a natural vertical to maintain a correct presentation of the visual image on the eye retina. In our study, the environment remained relatively unperturbed. However, the incorrectly oriented body character on the screen might be perceived as an environmental reference triggering reorganization of the participant's body alignment to fit with the frame. Deviation from a stability comfort zone due to the body shifting forward or backward could then destabilize posture.

Postural destabilization observed in altered viewing conditions could also be due to the conflict between the perceived proximity of the figure and the angle of actual optic axes. Cortical cells responsible for visual motion detection are sensitive to a specific axis of optic signal orientation (Movshon 1990). Distorted vertical presentation of the stimulus (the Mia character) could reduce sensitivity of these neurons and impair their ability to utilize visual stimulus for postural stabilization. This could then result in increased postural oscillations in viewing up and down conditions.

Surprisingly our results revealed only a modest and nonsignificant effect on the COG parameters when viewing and gaze angles were altered together. We were unable to replicate the findings of Buckley et al. (2005) and Fukushima et al. (2008), who showed that coordination of eye–head movements to view a target presented above or below eye level changed stance ground reaction forces. In our study the angular shifts of 25° were smaller than theirs, and so did not require a head movement. Indeed, our participants were instructed to keep their head still. Another possible explanation is that the combination of the effects of altering of gaze and viewing angles together resulted in a mutually compensating effect.

### Limitations of the study

This study has some limitations. Although we tried to dissociate the effects of gaze and viewing angles, no eye movements were recorded. We assumed that participants in our study followed instructions and altered eye position in different gaze conditions rather than use head movements. We also studied postural stability in relatively young healthy participants who had small-amplitude body oscillations during quiet stance. Altering the gaze and viewing angle may not have the same effect in individuals with postural control problems. We also studied a stationary rather than a moving target, so conclusions are limited to this. The effects could have been different with a moving, rather than stationary character. These issues will be addressed in further studies.

## Conclusion

Many neural mechanisms may be involved in postural reorganization due to changes in gaze and viewing angles. Those include proprioceptive feedback from extraocular muscles as they adjust eye position in the orbit and alterations in the output signal from the retina. The contribution of each of these mechanisms deserves systematic investigation. This study does not seek to these mechanisms, but instead provides evidence that viewing and gaze angles play different roles in the visual stabilization of upright posture. More research is needed to test whether similar mechanisms of visuomotor transformation are used when planning and executing postural other tasks as well voluntary goal-directed movements. Results of such research have potential uses in designing simulated environments to facilitate motor performance in such activities as teleoperation and functional rehabilitation.
